# A Study of Cutaneous Adverse Drug Reactions at a Tertiary Care Center in Andhra Pradesh, India

**DOI:** 10.7759/cureus.37596

**Published:** 2023-04-14

**Authors:** Shaik Ashifha, Jami Vijayashree, Kirankanth Vudayana, Dilipchandra Chintada, Pavani P, Pallavi G, Pooja Unnikrishnan

**Affiliations:** 1 Dermatology, Venereology and Leprosy, Great Eastern Medical School and Hospital, Srikakulam, IND

**Keywords:** scars, cadrs, acute generalised flexural exanthem, symmetrical drug related intertriginous and flexural exanthem, lichenoid drug eruption, erythroderma, toxic epidermal necrolysis, fixed drug eruption, antimicrobials

## Abstract

Introduction: Practically all physicians encounter a diverse range of suspected cutaneous adverse drug reactions (CADRs) in their daily clinical practice. The skin and mucosa are the most often encountered areas for the early presentation of numerous adverse drug reactions. Cutaneous adverse drug reactions are classified as benign or severe. The clinical manifestations of drug eruptions can range from mild maculopapular exanthema to severe cutaneous adverse drug reactions (SCARs).

Objective: To determine the varied clinical and morphological presentations of CADRs and to identify the culprit drug and common drugs causing CADRs.

Materials and methods: Patients with clinical features suspected of CADRs presenting to the outpatient department (OPD) of dermatology, venereology, and leprosy (DVL) between December 2021 to November 2022 at Great Eastern Medical School and Hospital (GEMS), Srikakulam, Andhra Pradesh, India, were considered for the study. This was a cross-sectional, observational study. The patient’s clinical history was taken in detail. This included chief complaints (symptoms, site of onset, duration, drug history, latency time between drug administration and the appearance of cutaneous lesions), family history, associated diseases, the morphology of lesions, and mucosal examination. Upon drug discontinuation, improvement in cutaneous lesions and systemic features were noted. A complete general examination, systemic examination, dermatological tests, and mucosal examination were performed.

Results: A total of 102 patients were involved in the study, of whom 55 were males and 47 were females. The male-to-female ratio was 1.17:1, with a slight male majority. The most common age group was 31 to 40 years for both males and females. Itching was the predominant complaint in 56 patients (54.9%). The mean latency period was shortest in urticaria (2.13+/- 0.99 hours) and longest in lichenoid drug eruption (4.33+/- 3.93 months). Most patients developed symptoms after a week of taking the drug (53.92%). A history of similar complaints was present in 38.23% of patients. Analgesics and antipyretics (39.2%) were the most common culprit drugs followed by antimicrobials (29.4%). Among analgesics and antipyretics, aceclofenac (24.5%) was the commonest culprit drug. Benign CADRs were observed in 89 patients (87.25%), and severe cutaneous adverse reactions (SCARs) were observed in 13 patients (12.74%). The common CADRs presented were drug-induced exanthem (27.4%). Imatinib-induced psoriasis vulgaris and lithium-induced scalp psoriasis were observed in one patient each. Severe cutaneous adverse reactions were observed in 13 patients (12.74%). Anticonvulsants, nonsteroidal anti-inflammatory drugs (NSAIDs), and antimicrobials were the culprit drugs for SCARs. Eosinophilia was present in three patients, deranged liver enzymes was present in nine patients, a deranged renal profile was present in seven patients, and death occurred in one patient with toxic epidermal necrolysis (TEN) of SCARs.

Conclusion: Before prescribing any drug to a patient, a detailed drug history and family history of drug reactions need to be obtained. Patients should be advised to avoid over-the-counter usage of medications and self-administration of drugs. If adverse drug reactions occur, it is advised to avoid readministration of the culprit drug. Drug cards must be prepared and given to the patient, mentioning the culprit drug as well as the cross-reacting drugs.

## Introduction

Physicians encounter various suspected cutaneous adverse drug reactions (CADRs) in their daily clinical practice. These CADRs are described as “an appreciably harmful or unpleasant reaction, resulting from an intervention related to the use of a medicinal product, which predicts risk from future administration and warrants prevention, specific treatment, alteration of the dosage regimen, or withdrawal of the product” [1,2]. The skin and mucosa are the most often encountered areas for the early presentation of numerous adverse drug reactions [3]. Around 2% to 3% of hospitalized patients have CADRs [4]. Cutaneous adverse drug reactions are classified as benign or severe. Although up to 2% of all adverse cutaneous medication eruptions are severe and potentially fatal, the vast majority of these reactions are benign [5]. Clinical manifestations of drug eruptions can range from mild maculopapular exanthema to severe cutaneous adverse drug reactions (SCARs), including Stevens-Johnson syndrome (SJS) and toxic epidermal necrolysis (TEN), which are uncommon but occasionally fatal. Other SCARs include drug reactions with eosinophilia and systemic symptoms (DRESS) [6]. Further allergological testing may be carried out to confirm the offending medication once the acute stage of the adverse response has been successfully treated [7]. To determine safer, alternative medications and to pinpoint the causes of cutaneous adverse drug responses, skin tests such as patches, pricks, and intradermal testing, are helpful [8]. Dermatologists are crucial in the diagnosis of these severe skin conditions [9]. Few published studies have examined the clinical aspects of drug reactions in India, and even less so in South India [10,11]. So, this study was conducted to determine the varied clinical and morphological presentations of CADRs and to identify the culprit drug and the common drugs causing CADRs.

## Materials and methods

In this cross-sectional observational study conducted at a tertiary care center in Srikakulam, Andhra Pradesh, India (approval no. 18/IEC/GEMS&H/2023), the participants' exposure and results were assessed simultaneously. After obtaining their written consent, all age groups and both sexes with clinical features suspected of CADRs who presented to the dermatology, venerology, and leprosy outpatient department or were referred from other departments between December 2021 and November 2022 at Great Eastern Medical School and Hospital (GEMS), were included. Drug toxicity, overdose, drug-drug interactions, intolerance, and non-immunologically predictable types of drug reactions such as pharmacological or secondary pharmacological side effects of a drug, were not included in this study.

The study comprised 102 patients who had reported experiencing varied cutaneous medication responses. The most important medications in relation to the typical skin symptoms and their importance to CADR were looked into. The clinical history of the patient was thoroughly recorded, and this included the chief complaints i.e., symptoms, site of onset, dosage, duration, indication, class of drug taken, latency time between drug administration, and the appearance of cutaneous lesions; family history; associated diseases; the morphology of lesions; sites on progression; mucosal examination; and associated systemic symptoms. Also observed were improvements in cutaneous lesions and systemic features upon drug discontinuation. In addition to the history of drug use, details about related allergies, comorbidities, and severity were noted.

A full physical examination was conducted, including a systemic assessment and dermatological examination such as morphology, site, the extent of involvement of the lesion, and mucosal examination. Digital images were captured. Hematological, biochemical, and viral indicators were investigated. The venereal disease research laboratory (VDRL) test was done when the underlying risk factors were present. When the diagnosis was in doubt, a histopathological examination of the biopsy was performed. When in doubt, dermatologic therapeutic outcomes, laboratory data, and clinicopathologic features were examined. After excluding other etiologies and illnesses with similar symptomatology, such as responses to certain foods, infections, and environmental variables, the diagnosis of CADR was made. According to the Naranjo Adverse Drug Reaction Probability Scale, the causality of CADRs was evaluated, and CADRs were rated as extremely probable (definite), possible, and probable. Throughout the course of the study, the data was gathered, examined, tallied, and conclusions were drawn through mean +/- standard deviation (SD) by using MS Excel (Microsoft Corp., Redmond, WA, USA). To prevent any future accidents, all the patients were informed about CDRs and given a list of medications that could cause reactions.

## Results

This study included a total of 102 patients out of which 55 were males and 47 were females. The most common age range was 31 to 40 years in both genders (Table 1). 

**Table 1:** Age distribution of patients

Age (in years)	Female	Male	Total
≤20	3 (6.3%)	5 (9%)	8 (7.8%)
21-30	8 (17.0%)	8 (14.5%)	16 (15.6%)
31-40	16 (34.0%)	17 (30.9%)	33 (32.3%)
41-50	12 (25.5%)	14 (25.4%)	26 (25.4%)
51-60	4 (8.5%)	7 (12.7%)	11 (10.7%)
>60	4 (8.5%)	4 (7.2%)	8 (7.8%)
Total	47 (100%)	55 (100%)	102 (100%)

The predominant complaint was that of itching in 56 patients (54.9%). The latency period, i.e., the time period between drug administration and the appearance of eruptions on the skin, was recorded. The mean latency period was the shortest in urticaria and fixed drug eruption (FDE) (2.13+/- 0.99 hours and 2.23+/- 1.13 hours, respectively), followed by angioedema (5.33+/- 1.15 hours). The longest latency period was observed in a lichenoid drug eruption (4.33 +/- 3.93 months). Most of the patients developed symptoms after a week of taking the drug: 55 patients (53.92%), 41 patients (40.19%) in a week, and six patients (5.88%) more than a month. 

A history of atopy, either individual or familial, was present in 6.8% of patients. Also present was a history of similar complaints in 38.23% of patients. A definite history of using the same drug previously with similar adverse reactions was given by 23.5% of patients.

Multiple drug usage was noticed in 61.7% of patients; antibiotics and analgesics were commonly used together. Analgesics and antipyretics (39.2%) were the most common culprit drugs, followed by antimicrobials (29.4%) and anticonvulsants (10.7%). Among analgesics and antipyretics, aceclofenac (24.5%) was the most frequent culprit drug, followed by paracetamol (9.8%). In antimicrobials, the penicillin group (15.6%) was the most common. Amoxicillin (11.7%) was most commonly used in the penicillin group. In anticonvulsants, phenytoin (4.9%) and carbamazepine (5.8%) were used by almost the same percentage of patients.

**Figure 1:**
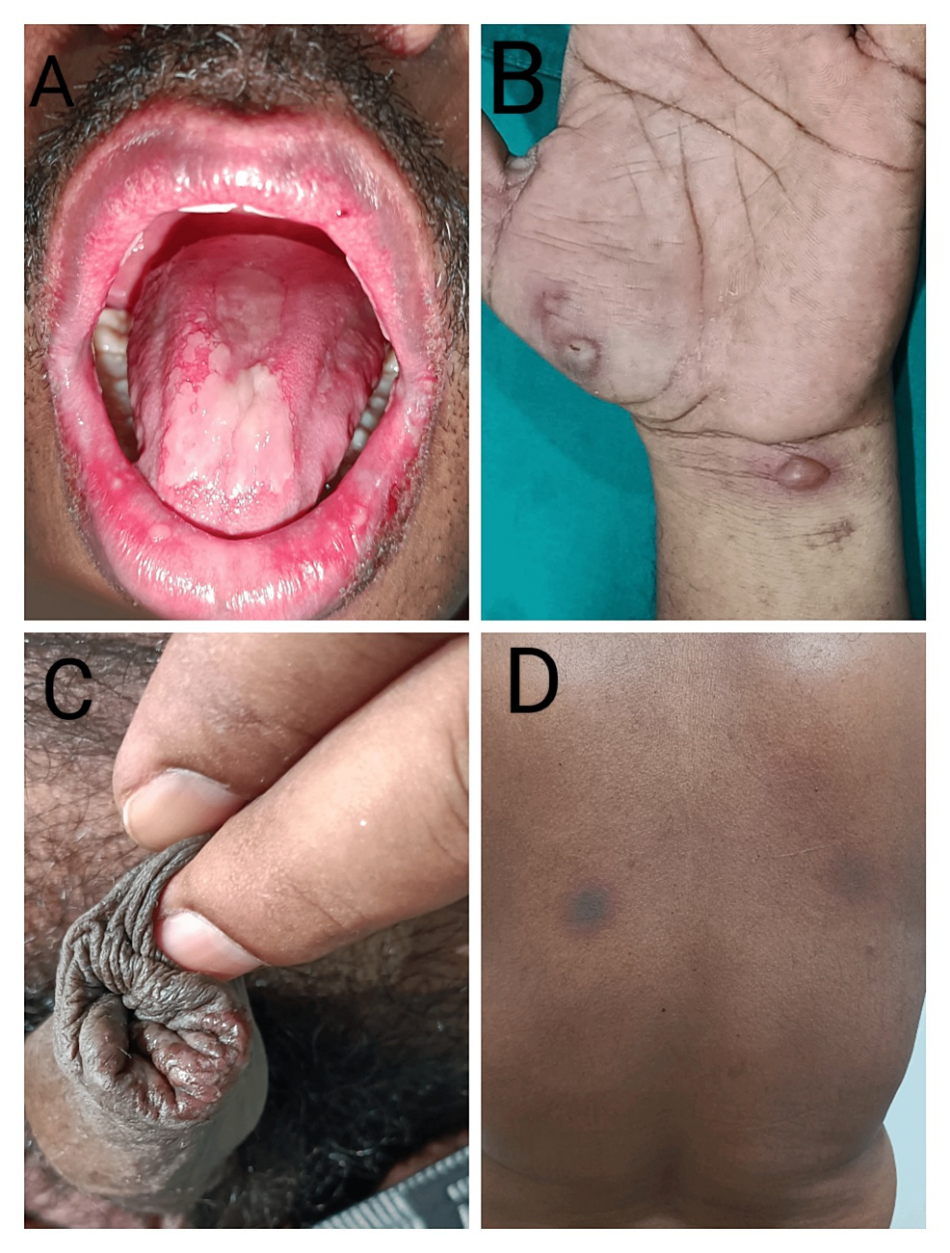
Fixed drug eruption secondary to NSAIDs A: Oral erosions, B: Bullae over the left wrist, C: Genital erosions, D: Hyperpigmentation NSAIDs: Nonsteroidal anti-inflammatory drugs

**Figure 2:**
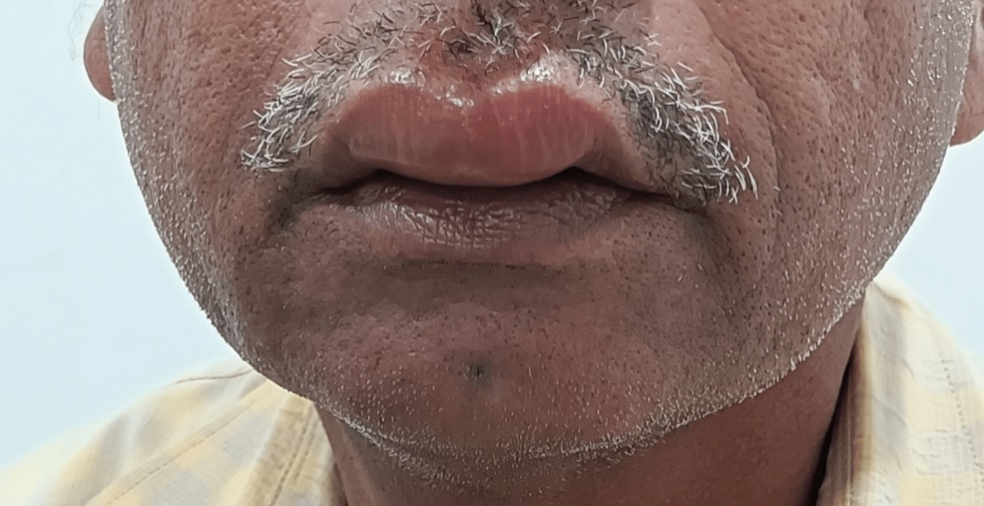
Drug-induced angioedema secondary to cefixime

**Figure 3:**
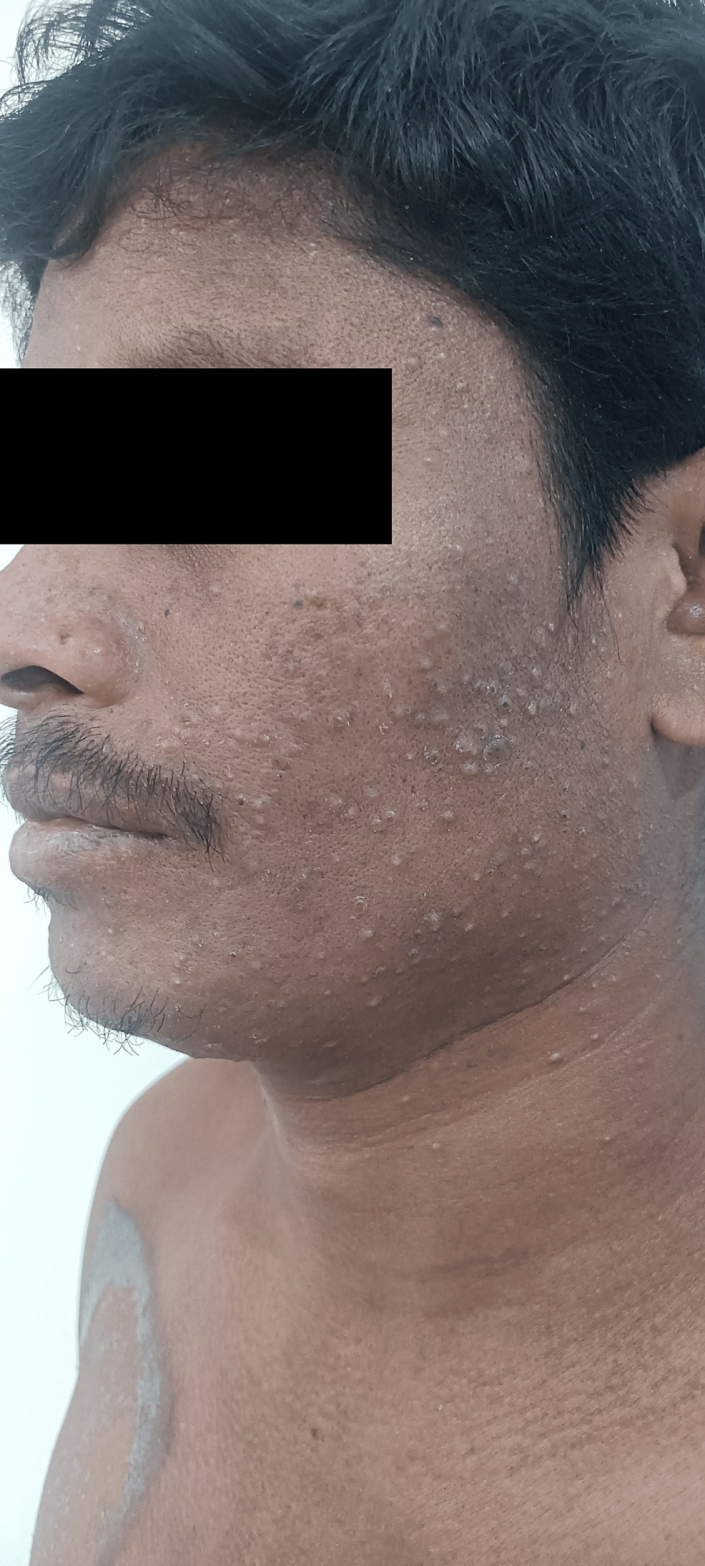
Sudden onset papulopustular rash (acneiform eruption) in a patient on corticosteroids

**Figure 4:**
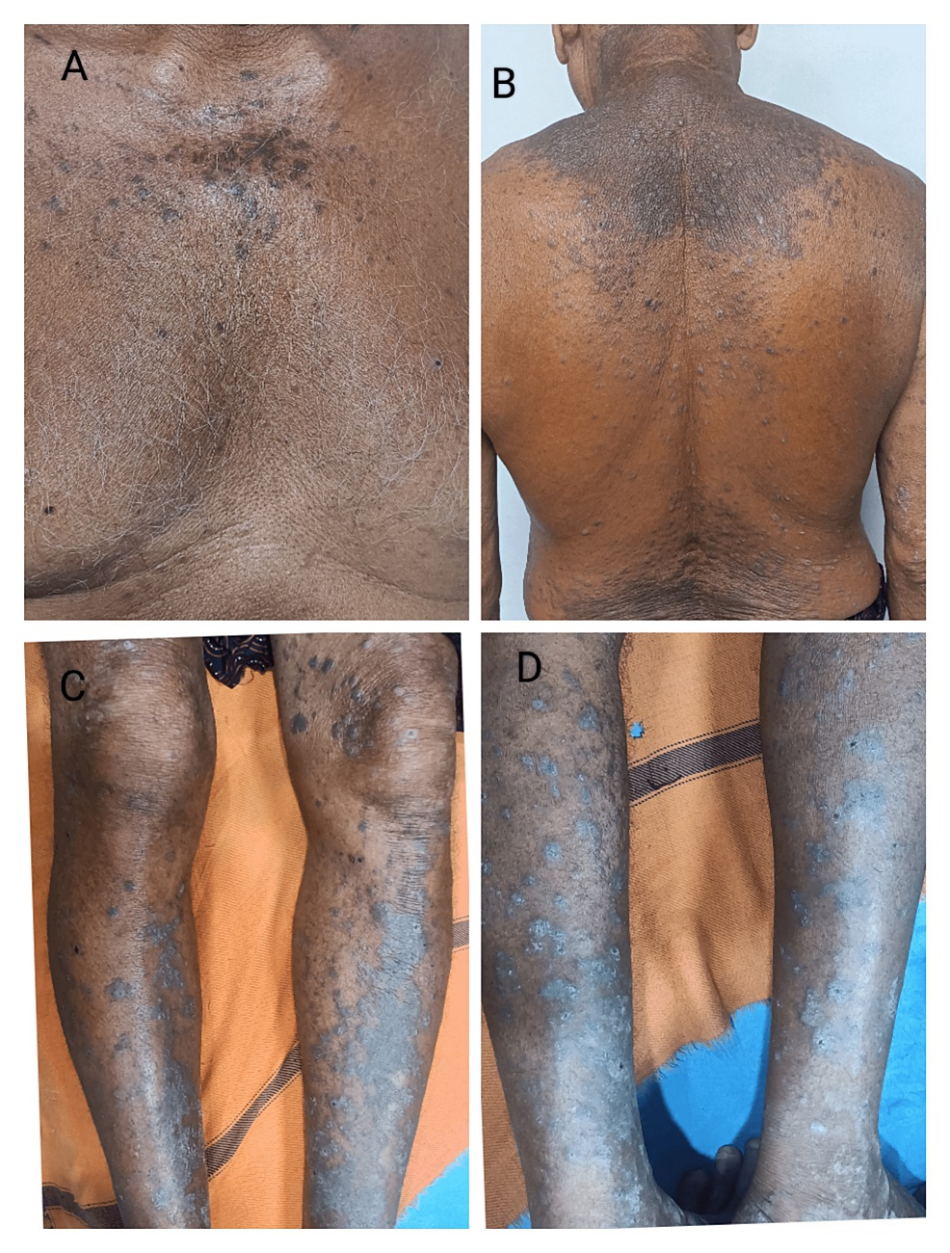
Lichenoid drug eruption due to anti-tubercular treatment; multiple, hyperpigmented to violaceous plaques distributed all over A: Chest, B: Back, C: Extensor aspect of the lower limb, D: Flexor aspect of the lower limb

Benign CADRs were observed in 89 patients (87.25%), and SCARs were observed in 13 patients (12.74%). The common CADRs presented in this study were drug-induced exanthem (27.4%), FDE (20.5%), and drug-induced urticaria (14.7%) (Table 2). Analgesics and antipyretics were common causes of FDE (10.7%) (Figure 1), drug-induced exanthem (8.8%), and drug-induced urticaria (6.8%). Antimicrobials were the common cause of drug-induced exanthem (8.8%), FDE (7.8%), and drug-induced urticaria (5.8%). Cephalosporins caused reactions in 10.7% of patients, commonly with ceftriaxone and cefixime. In cephalosporins, ceftriaxone has caused FDE, cefixime has caused angioedema (Figure 2), and cephalexin has caused urticaria. Bullous FDE was commonly observed. Antimicrobials causing FDE were ceftriaxone, cefixime, metronidazole, amoxicillin, and co-trimoxazole. Corticosteroids caused an acneiform eruption (7.8%) (Figure 3). Omeprazole caused urticaria and erythroderma in one patient each. Anti-tubercular drugs caused lichenoid drug eruption in two patients (Figure 4) and erythroderma in one patient. Mucosal involvement was seen in 40% of patients.

**Table 2:** Distribution of common drugs and mean latency period of various benign CADRs ATT: Anti-tubercular treatment; PCM: Paracetamol; SDRIFE: Symmetrical drug-related intertriginous and flexural exanthem; NSAIDs: Nonsteroidal anti-inflammatory drugs, CADRS: Cutaneous adverse drug reactions

Benign CADRs	Frequency	Common Drug	Mean Latency Period
Exanthematous drug reaction	28 (31.4%)	NSAIDs (12)	3.07+/- 2.41 days
Fixed drug eruption	21 (23.5%)	Analgesics (11)	2.23+/- 1.13 hours
Urticaria	15 (16.8%)	NSAIDs (7)	2.13 +/- 0.99 hours
Acneiform eruption	11 (12.3%)	Corticosteroids (8)	3.09+/- 1.04 weeks
Lichenoid drug eruption	6 (6.7%)	NSAIDs (2)	4.33+/- 3.93 months
Angioedema	3 (3.3%)	Antimicrobials (2)	5.33 +/- 1.15 hours
Pruritus	2 (2.2%)	NSAIDs (5)	6 +/- 1.41 days
Drug-induced psoriasis	2 (2.2%)	Imatinib & Lithium	9 +/- 1.41 days
SDRIFE	1 (1.1%)	NSAIDs (1)	2 days
Total (89)	89 (100%)	-	-

Imatinib-induced psoriasis vulgaris was observed in a patient with leukemia, and lithium-induced scalp psoriasis was observed in a patient with bipolar disorder. There was no personal, past, or family history of psoriasis in these two patients. On stopping the offending drug, psoriasis resolved without any treatment. Alternate drugs were given to these patients. 

Severe cutaneous adverse drug reactions were observed in 13 patients (12.74%), as shown in Table 3. Out of them, six were female and seven were male. Anticonvulsants, NSAIDs, and antimicrobials were the culprit drugs for SCARs. The latency period was shortest for acute generalized exanthematous pustulosis (AGEP), i.e., two days, caused by aceclofenac; and longest for DRESS (20 days due to anti-tubercular treatment). The mean latency period was the shortest for AGEP (2.5 days). 

**Table 3:** The frequency and most common drug causing SCARs, and the mean latency period in various SCARs SCARs: Severe cutaneous adverse drug reactions; TEN: Toxic epidermal necrolysis; AGEP: Acute generalized exanthematous pustulosis; SJS: Stevens-Johnson syndrome; DRESS: Drug-related eosinophilia with systemic symptoms

SCARs	Frequency	Common Drug	Mean Latency Period
Erythroderma	6 (46.1%)	Anticonvulsants (2)	11.66 +/- 3.61 days
TEN	3 (23.0%)	Anticonvulsants (2)	8 +/- 1.73 days
AGEP	2 (15.3%)	Amoxicillin(1)	2.5 +/- 0.7 days
SJS	1 (7.6%)	Co-trimoxazole (1)	10 days
DRESS	1 (7.6%)	Ibuprofen (1)	20 days
Total	13 (100%)	-	-

**Figure 5:**
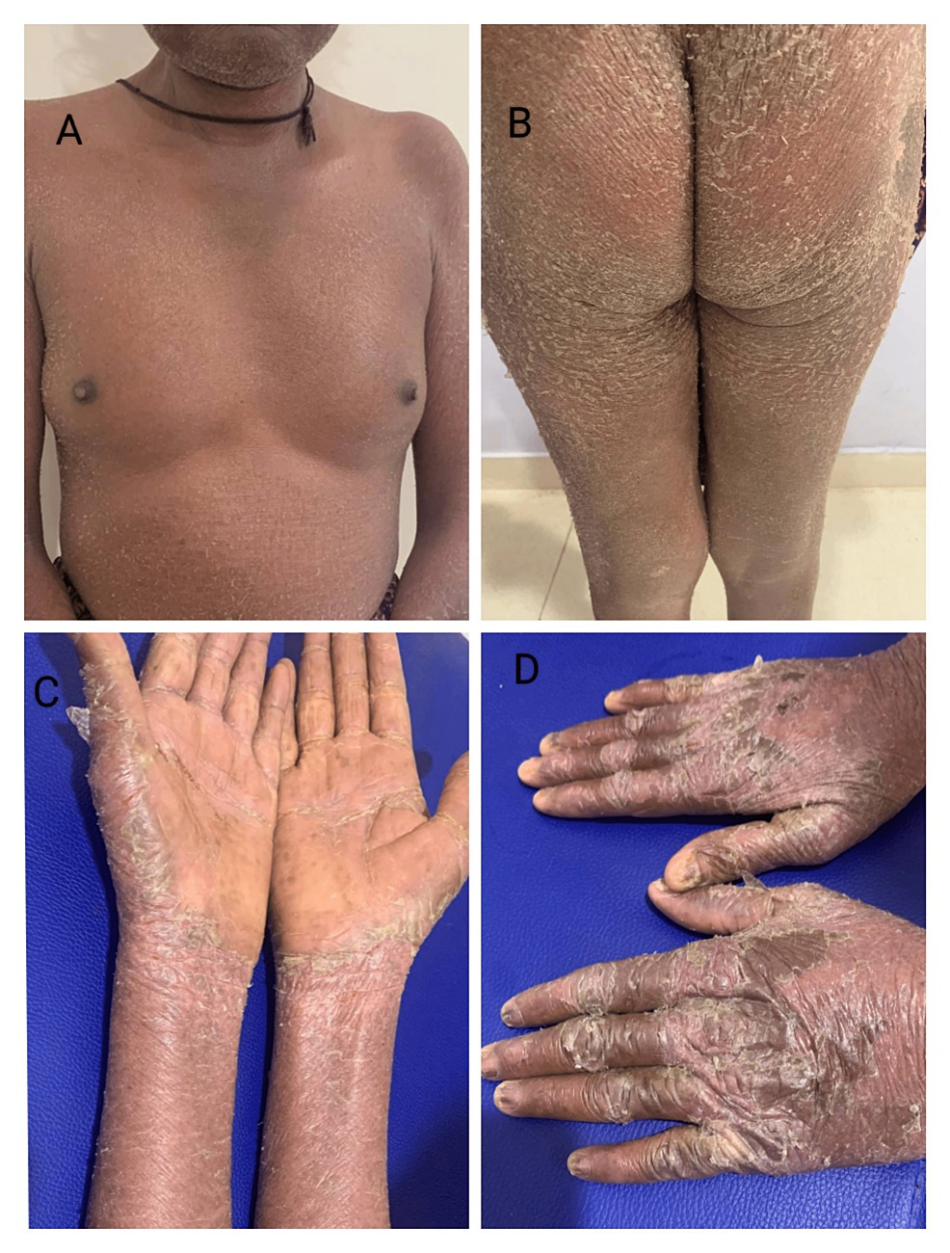
Erythroderma secondary to NSAIDs A: Trunk, B: Lower limb, C: Forearm and palms, D: Dorsal aspect of hands NSAIDs: Nonsteroidal anti-inflammatory drugs

**Figure 6:**
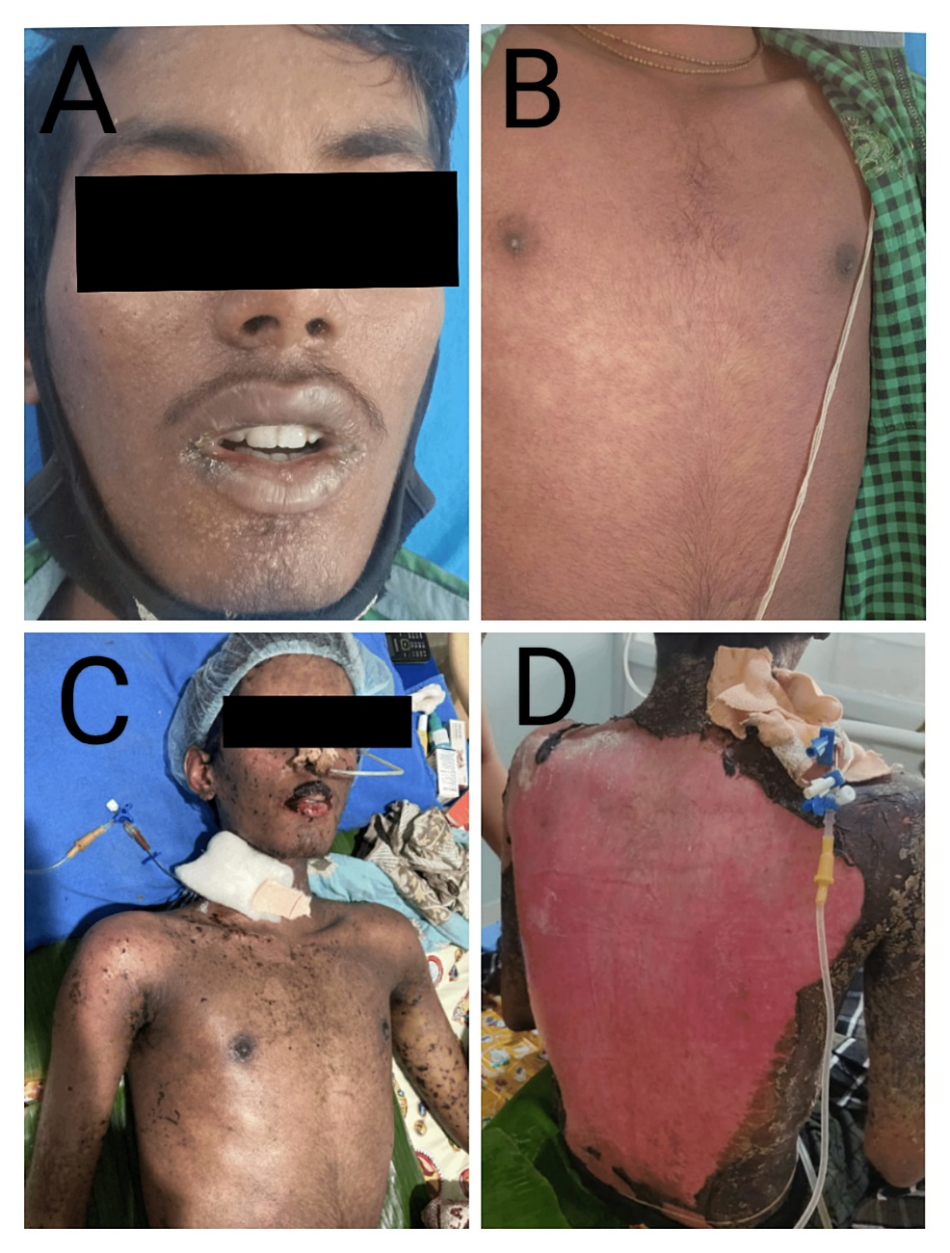
Toxic epidermal necrolysis due to phenytoin A, B: On the first day of the presentation, C: Fifth day of the presentation, D: Seventh day of the presentation

Erythroderma was the most common SCAR seen in six patients (Figure 5). A male patient aged 18 who had TEN (Figure 6) was the youngest patient with SCARs secondary to phenytoin and had used phenytoin for myotonias. The reaction time for two out of three TEN cases was 14 days, caused by phenytoin and carbamazepine. Toxic epidermal necrolysis in the third patient occurred due to NSAIDs, with a reaction time of 12 days.

Out of the total patients in the study, three had eosinophilia, nine had deranged liver enzymes, seven presented with a deranged renal profile, and death occurred in one patient (TEN) of SCARs due to sepsis. The Naranjo adverse drug reaction probability scale for assessment of causality shows probable association in 85.29%, definite association in 10.78%, and possible association in 3.92% of patients. As a result of these reactions, drug withdrawal was advised for all patients.

## Discussion

In this study, the male-to-female ratio was 1.17:1, with a slight male majority. This correlates with Patel (&) Marfatia's study [11], Rajendran et al. [12], and Jha et al. [13], but does not correlate with Pudukadan (&) Thappa's study [10], which shows a female majority. The average age range of this study was 31 to 40 years in both genders, followed by 41 to 50 years. This is consistent with Sharma et al.'s study [14], in which the majority of patients were between the ages of 20 and 39. In the study conducted by Rajendran et al. [12], the most common age group was 40 to 60 years.

The predominant complaint of itching (54.9%) correlates with the Rajendran et al. [12] study. Atopy history in patients or their family members was present in 6.8%, which is very low compared to the Al‑Raiee et al. [15] study, which shows 21%.

The common CADRs presented in this study were drug-induced exanthem (27.4%) and FDE (20.5%), which are similar to the studies by Rajendran et al. [12], Jha et al. [13], and Saha et al. [16]. It doesn’t correlate with Al-Raiee et al. [15] and Chatterjee et al. [17], in which drug-induced urticaria was the most common presentation. In Pudukadan (&) Thappa’s study [10], fixed drug eruptions were the most common presentation. The correlation of drug reactions with other studies has been shown in Table 4.

**Table 4:** CADRs patterns comparison with previous studies CADRs: cutaneous adverse drug reactions; FDE: fixed drug eruption; TEN: toxic epidermal necrolysis; SJS: Stevens-Johnson syndrome

CADRs	Current study	Rajendran et al. [12]	Jha et al. [13]	Al‑Raiee et al. [15]	Saha et al. [16]	Chatterjee et al. [17]
Exanthem	27.4	31.5	42.6	13	30.1	25.4
FDE	20.5	13.4	9.3	14	24.5	25.2
Urticaria & angioedema	17.6	8.8	30.2	41	5.6	27.2
Erythroderma	5.8	0.9	0.77	4	7.54	-
TEN, SJS	3.9	6.9	2.32	9	24.5	1.62
Others	24.5	32	11.32	13	-	18

Analgesics and antipyretics (39.2%) were the most common culprit drugs, followed by antimicrobials (29.4%) and anticonvulsants (10.7%). This correlates with the Al-Raiee et al. [15] study, in which NSAIDs are the common culprit drugs, but does not correlate with the Pudukadan(&) Thappa study [10], which shows antimicrobials as the most common culprit drug. Culprit drug correlation with other studies has been shown in Table 5.

**Table 5:** Culprit drug comparison with previous studies NSAIDs: Nonsteroidal anti-inflammatory drugs

Culprit Drug	Current study	Rajendran et al. [12]	Jha et al. [13]	Al‑Raiee et al. [15]	Saha et al. [16]	Chatterjee et al. [17]
NSAIDs	29.4	7.9	15.5	29	11.3	21.51
Antimicrobials	29.4	30.1	64.73	29	50.9	34.1
Anticonvulsants	10.7	18.1	7.36	8	11.3	32.88
Others	41.1	43.9	12.84	34	26.4	11.5

Severe cutaneous adverse drug reactions were observed in 13 patients (12.74%); this correlates with the Sasidharanpillai et al. [18] study (13.20%) but does not correlate with the Saha et al. [16] study (32.04%) and Choon and Lai et al. [19] study (39.7%), which shows a higher incidence. Neither does this correlate with the study by Al-Raiee et al. [15], where 25 out of 100 patients presenting with CADRs had SCARs, which is high compared to this study. In this study, deranged liver enzymes (8.8%) and a deranged renal profile (6.8%) were low, and similar to Jha et al.'s study [13]. Erythroderma was the most common SCAR observed in this study, in contrast to Jha et al.'s study [13] showing SJS-TEN as the most common SCAR.

The reaction time to phenytoin-induced TEN was observed to be 14 days; this did not correlate with Al-Quteimat 's study [20] where the acute phase lasts from eight to 12 days. The latency period for DRESS was the longest at 20 days and correlated with the study conducted by Husain et al. [21] which mentioned DRESS has a later onset and longer duration than other drug reactions, with a latent period of two to six weeks

The longest latency period was observed in lichenoid drug eruption (4.33 +/- 3.93 months), which correlated with Maul et al.'s study [22], which found a 15.7-week latency between drug initiation and the manifestation of cutaneous lichenoid drug eruptions. Anticonvulsants were found to be the common drug causing erythroderma, which did not correlate with a study conducted by Tan et al. [23] where cases of drug-related erythroderma, traditional medications, and antituberculous medications are common causes. The most common anticonvulsants causing SCARs are phenytoin and carbamazepine which correlated with a study conducted by Singh et al. [24], which found serious CADR like TEN and SJS to be more likely in patients prescribed phenytoin and carbamazepine.

Limitations

A drug rechallenge was not performed. In vitro tests for confirmation were not done due to in vitro and in vivo discordance in the results and the limited availability of in vitro tests.

## Conclusions

Out of the total patients attending the outpatient department, 102 patients presented with CADRs, thus the incidence is 0.97%. In this study, benign CADRs were commonly seen compared to SCARs. Antimicrobials and NSAIDs are the most common culprit drugs which also correlates with existing literature. Anticonvulsants are the most common culprit drugs in SCARs. Patients with a history of SCARs must be advised to avoid the drug completely in the future due to systemic involvement in SCARs leading to morbidity and mortality. Awareness about CADRs in the general population is also required to stop polypharmacy which hinders the identification of the culprit drug, since drug rechallenge in SCARs may lead to complications and limited availability of in vitro tests. Few drug reactions have a longer latency period, in which there is a risk of delayed diagnosis or misdiagnosis, thus thorough knowledge of CADRs is required.

Cutaneous adverse drug reactions can vary from mild reactions to severe, life-threatening, or even fatal reactions. Drug histories and family histories of drug reactions need to be enquired about in all patients before prescribing any medication. Thus, whenever a new drug is given to a patient, the physician needs to be cautious and monitor the patient vigilantly. And also, patients should be advised to avoid over-the-counter usage of medications and self-administration of drugs. If adverse drug reactions occur, it is advised to avoid readministration of the culprit drugs. It is essential to prepare drug cards for the patient, mentioning both the culprit drug and cross-reacting drugs. Early identification of different morphological characteristics is crucial for identifying the culprit drug and stopping it right away to prevent iatrogenic morbidity and mortality.

## References

[1] Hussain R, Hassali MA, Ur Rehman A, Muneswarao J, Hashmi F (2020). Physicians' understanding and practices of pharmacovigilance: qualitative experience from a lower middle-income country. Int J Environ Res Public Health.

[2] Coleman JJ, Pontefract SK (2016). Adverse drug reactions. Clin Med (Lond).

[3] Zhang S, Liu XY, Zhang JZ, Cai L, Zhou C (2019). Drug-induced toxic epidermal necrolysis with secondary Aspergillus fumigatus infection: a case report. Beijing Da Xue Xue Bao Yi Xue Ban.

[4] Rana S, Gupta K, Agarwal N, Ahamed AN (2021). A study of cutaneous adverse drug reactions and their association with autoimmune diseases at a tertiary centre in South-West Rajasthan, India. Indian J Dermatol.

[5] Hoetzenecker W, Nägeli M, Mehra ET (2016). Adverse cutaneous drug eruptions: current understanding. Semin Immunopathol.

[6] Chung WH, Wang CW, Dao RL (2016). Severe cutaneous adverse drug reactions. J Dermatol.

[7] Mockenhaupt M (2022). Drug allergy and cutaneous adverse reactions. Handb Exp Pharmacol.

[8] Barbaud A, Castagna J, Soria A (2022). Skin tests in the work-up of cutaneous adverse drug reactions: a review and update. Contact Dermatitis.

[9] Reyes M, Kortepeter C, Muñoz M (2022). Postmarket assessment for drugs and biologics used in dermatology and cutaneous adverse drug reactions. Dermatol Clin.

[10] Pudukadan D, Thappa DM (2004). Adverse cutaneous drug reactions: clinical pattern and causative agents in a tertiary care center in South India. Indian J Dermatol Venereol Leprol.

[11] Patel RM, Marfatia YS (2008). Clinical study of cutaneous drug eruptions in 200 patients. Indian J Dermatol Venereol Leprol.

[12] Rajendran L, Thyvalappil A, Sridharan R, Ajayakumar S, Deep S, Divakaran B (2021). A study of cutaneous adverse drug reactions in a tertiary care center in South India. Clin Dermatol Rev.

[13] Jha N, Alexander E, Kanish B, Badyal DK (2018). A study of cutaneous adverse drug reactions in a tertiary care center in Punjab. Indian Dermatol Online J.

[14] Sharma R, Dogra D, Dogra N (2015). A study of cutaneous adverse drug reactions at a tertiary center in Jammu, India. Indian Dermatol Online J.

[15] Al-Raaie F, Banodkar DD (2008). Epidemiological study of cutaneous adverse drug reactions in Oman. Oman Med J.

[16] Saha A, Das NK, Hazra A, Gharami RC, Chowdhury SN, Datta PK (2012). Cutaneous adverse drug reaction profile in a tertiary care out patient setting in eastern India. Indian J Pharmacol.

[17] Chatterjee S, Ghosh AP, Barbhuiya J, Dey SK (2006). Adverse cutaneous drug reactions: a one year survey at a dermatology outpatient clinic of a tertiary care hospital. Indian J Pharmacol.

[18] Sasidharanpillai S, Riyaz N, Khader A, Rajan U, Binitha MP, Sureshan DN (2015). Severe cutaneous adverse drug reactions: a clinicoepidemiological study. Indian J Dermatol.

[19] Choon SE, Lai NM (2012). An epidemiological and clinical analysis of cutaneous adverse drug reactions seen in a tertiary hospital in Johor, Malaysia. Indian J Dermatol Venereol Leprol.

[20] Al-Quteimat OM (2016). Phenytoin-induced toxic epidermal necrolysis: review and recommendations. J Pharmacol Pharmacother.

[21] Husain Z, Reddy BY, Schwartz RA (2013). DRESS syndrome: Part I. Clinical perspectives. J Am Acad Dermatol.

[22] Maul JT, Guillet C, Oschmann A (2023). Cutaneous lichenoid drug eruptions: a narrative review evaluating demographics, clinical features and culprit medications. J Eur Acad Dermatol Venereol.

[23] Tan GF, Kong YL, Tan AS, Tey HL (2014). Causes and features of erythroderma. Ann Acad Med Singap.

[24] Singh PK, Kumar MK, Kumar D, Kumar P (2015). Morphological pattern of cutaneous adverse drug reactions due to antiepileptic drugs in Eastern India. J Clin Diagn Res.

